# Increased deposition of C3b on red cells with low CR1 and CD55 in a malaria-endemic region of western Kenya: Implications for the development of severe anemia

**DOI:** 10.1186/1741-7015-6-23

**Published:** 2008-08-21

**Authors:** Collins O Odhiambo, Walter Otieno, Christine Adhiambo, Michael M Odera, José A Stoute

**Affiliations:** 1 The US Army Medical Research Unit and the Kenya Medical Research Institute, Nairobi, Kenya; 2 Department of Medicine, the Uniformed Services University of the Health Sciences, Bethesda, Maryland, USA; 3 Division of Malaria Vaccine Development, Department of Cellular Injury, The Walter Reed Army Institute of Research, Robert Grant Avenue, Silver Spring, MD 20910, USA; 4 Pennsylvania State College of Medicine, 500 University Drive, MC: H036, Rm C6833, Hershey, PA 17033, USA

## Abstract

**Background:**

Severe anemia due to *Plasmodium falciparum *malaria is a major cause of mortality among young children in western Kenya. The factors that lead to the age-specific incidence of this anemia are unknown. Previous studies have shown an age-related expression of red cell complement regulatory proteins, which protect erythrocytes from autologous complement attack and destruction. Our primary objective was to determine whether in a malaria-endemic area red cells with low levels of complement regulatory proteins are at increased risk for complement (C3b) deposition *in vivo*. Secondarily, we studied the relationship between red cell complement regulatory protein levels and hemoglobin levels.

**Methods:**

Three hundred and forty-two life-long residents of a malaria-holoendemic region of western Kenya were enrolled in a cross-sectional study and stratified by age. We measured red cell C3b, CR1, CD55, and immune complex binding capacity by flow cytometry. Individuals who were positive for malaria were treated and blood was collected when they were free of parasitemia. Analysis of variance was used to identify independent variables associated with the %C3b-positive red cells and the hemoglobin level.

**Results:**

Individuals between the ages of 6 and 36 months had the lowest red cell CR1, highest %C3b-positive red cells, and highest parasite density. Malaria prevalence also reached its peak within this age group. Among children ≤ 24 months of age the %C3b-positive red cells was usually higher in individuals who were treated for malaria than in uninfected individuals with similarly low red cell CR1 and CD55. The variables that most strongly influenced the %C3b-positive red cells were age, malaria status, and red cell CD55 level. Although it did not reach statistical significance, red cell CR1 was more important than red cell CD55 among individuals treated for malaria. The variables that most strongly influenced the hemoglobin level were age, the %C3b-positive red cells, red cell CR1, and red cell CD55.

**Conclusion:**

Increasing malaria prevalence among children >6 to ≤ 36 months of age in western Kenya, together with low red cell CR1 and CD55 levels, results in increased C3b deposition on red cells and low hemoglobin. The strong contribution of age to C3b deposition suggests that there are still additional unidentified age-related factors that increase the susceptibility of red cells to C3b deposition and destruction.

## Background

*Plasmodium falciparum *malaria is responsible for 1 to 2 million deaths per year, with most in sub-Saharan Africa [1]. One unexplained but consistent feature of the epidemiology of clinical malaria is the age distribution of syndromes of severe disease. Severe anemia is most common in areas of intense transmission of *P. falciparum *and tends to occur in children <3 years of age, whereas cerebral malaria occurs in areas where the annual inoculation rate is low, and it occurs in older children and adults [2-5].

The pathogenesis of severe anemia in malaria is complex. However, a number of observations suggest that the destruction of uninfected red cells is a significant contributor to, if not the major cause of, this anemia: uninfected red cells have a decreased life span in patients with *P. falciparum *[6] and the hematocrit can continue to decrease for days following treatment [7]. Further, the fact that the life span of erythrocytes is 120 days suggests that bone marrow dysfunction would have to be very prolonged to make a significant contribution to this anemia. A mathematical model of severe malarial anemia has revealed that with each lysed infected erythrocyte a further 8.5 uninfected erythrocytes are destroyed [8]. These observations suggest that destruction of uninfected red cells takes place during malaria infection. Complement activation and its deposition on red cells are prime suspects in this process. Complement is activated during malaria infection [9,10] and C3d and IgG, molecules that are implicated in the removal of senescent red cells via erythrophagocytosis [11], have been detected on red cells of children with severe malaria [12,13].

In order to understand the nature of the susceptibility of red cells to complement activation during malaria infection we have investigated the expression of red cell complement regulatory proteins in persons living in a malaria-endemic area. Complement receptor 1 (CR1, CD35) is a 200 kDa protein that is found on red cells and most leukocytes [14]. It also accelerates the decay of C3 and C5 convertases, protein complexes that catalyze the cleavage of C3 and C5 into C3b and C5b respectively [15]. Red cells are able to bind C3b-bearing immune complexes (ICs) via CR1 and carry them to the liver and spleen where they are removed from circulation [16,17]. Therefore, CR1 prevents the deposition of C3b on cell surfaces and has a critical role in the removal of ICs from circulation.

The other red cell complement regulatory protein that we have studied is decay accelerating factor (DAF, CD55) [18]. It is a 70 kDa protein linked to the cell membrane via glycosyl-phosphatidyl-inositol (GPI). CD55 catalyzes the degradation of C3 convertases and also promotes the inactivation of C3b [19,20]. Deficiency in membrane DAF and CD59, a third red cell complement regulator, leads to paroxysmal nocturnal hemoglobinuria, a condition characterized by massive hemolysis due to complement activation on red cells. Consequently, red cell complement regulatory proteins may play an important role in protecting red cells from complement-mediated destruction as a result of IC formation and complement activation that occur during malaria infection [9,21]. Consistent with this hypothesis, we have found that red cells of children with *P. falciparum *infection and severe anemia have acquired deficiencies in CR1 and CD55, and increased C3b deposition [13,22]. Excessive C3b deposition on red cells during malaria infection as a result of deficiency in red cell complement regulatory proteins may result in increased destruction of red cells by phagocytosis and severe anemia [15].

To understand the age predilection of severe anemia during *P. falciparum *infection, we have studied the relationship between red cell CR1 and CD55 and age. Our studies suggest that the expression of red cell CR1 and CD55 are age-dependent, decreasing from birth to a nadir somewhere between 1 and 2 years of age and rising thereafter [23]. This pattern has been observed in populations of diverse ethnic origin and is independent of malaria infection. We have proposed that the changes in the expression of red cell complement regulatory proteins with age may influence the susceptibility of red cells to complement-mediated damage and, by extension, the age predilection for severe anemia during malaria infection [24]. To test whether the susceptibility of red cells to C3b deposition is influenced by the age-related changes in complement regulatory proteins we carried out a cross-sectional study of individuals in a malaria-holoendemic region of western Kenya, measured their red cell CR1, CD55, and IC binding capacity, and determined the %C3b-positive red cells.

## Methods

### Study design and population

The study was cross-sectional in design and was approved by the Walter Reed Army Institute of Research Human Use Research Committee and by the National Ethical Review Committee, Nairobi, Kenya. It was carried out from October through December 2004 in Kombewa Division, an area off the shores of Lake Victoria within Kisumu District. Malaria is holoendemic in this region, occurring throughout the year with peak seasons during the long rains (March through August) and during the short rains (October through December) [25]. The area is divided into four sub-Divisions and 12 political locations with approximately 62,000 inhabitants. The majority of the inhabitants are of the Luo ethnic group and dedicate themselves to subsistence farming and fishing.

Enrollment was stratified into nine age groups selected based on the age distribution of children with severe anemia (Hgb ≤ 5.0 g/dl) and *P. falciparum *malaria from Kisumu District, western Kenya, who participated in our studies from 1998 to 2001 [13,26,27]: 0 to ≤ 6 months, >6 to ≤ 12 months, >12 to ≤ 18 months, >18 to ≤ 24 months, >24 to ≤ 36 months, >36 to ≤ 72 months, >72 to ≤ 144 months, >144 to ≤ 288 months, and >288 to ≤ 540 months. Since we had no previous data on the levels of C3b deposition on red cells, enrollment targets for each group were set to maximize the probability of detecting significant differences between the >6 to ≤ 12 month group, where we had seen the lowest level of CR1 in a previous study [28], and other groups. The inclusion criterion was any person male or female of age 45 years or younger who was a life-long resident of the study area. As many conditions including malaria [13], HIV infection [29], and others [30,31] can alter the level of red cell complement regulatory proteins, the following exclusion criteria were established: 1) evidence on clinical grounds of malnutrition manifested by marasmus or kwashiorkor; 2) immunocompromised status manifested by weight loss, thrush, or diffuse adenopathy; 3) severe anemia (Hgb ≤ 5.0 g/dl); 4) bacterial infection (for example, pneumonia); 5) malignancy; and 6) blood transfusion within 3 months preceding the study.

A preliminary census was carried out within selected villages to identify potential participants. All individuals who met the entry criteria were invited to participate. On the days of recruitment individuals or parents with children self-reported to the clinic for enrollment in the study. Informed consent was obtained prior to any procedures. Individuals underwent a standard medical evaluation. Date of birth was confirmed by national registration card, baptismal certificate, or birth certificate. During the initial screening, blood obtained by finger prick was used to prepare thick and thin Giemsa-stained blood smears. If an individual was positive for malaria, he or she was treated with artemether/lumefantrine [32] and asked to return 2 weeks later for re-evaluation. Individuals who were febrile (oral or axillary temperature >37.5°C) without malaria were evaluated and treated for any minor illness identified and asked to return 2 weeks later. Individuals who returned for re-evaluation were screened for malaria again. If found to be malaria positive or febrile at this second screen, they were again evaluated, treated, and asked to come back 2 weeks later.

### Blood samples and smears

Malaria smears were read by trained and certified microscopists. All smears were read in duplicate by independent microscopists who were unaware of the status of the participants. Discrepancies were settled by a third independent microscopist. A smear required a minimum of 200 high power fields scanned prior to being considered negative. Once an individual was deemed well and asymptomatic, a 2.5 ml ethylenediaminetetraacetic acid (EDTA)-anticoagulated sample of venous blood was obtained. An aliquot was used for complete blood count determination using an automated hematology analyzer (Coulter, Hialeah, FL). The remaining sample was centrifuged and the plasma removed. The red blood cell pellet was then cryopreserved until used [33].

### Flow cytometric measurement of CR1 and CD55

Erythrocyte CR1 and CD55 levels were determined using frozen samples. In preliminary experiments, we observed no difference in the level of red cell complement regulatory proteins between fresh and frozen samples. All primary antibodies were titered to saturation. 10 μl of thawed erythrocyte pellet was washed twice in 1 ml of Alsever's buffer and resuspended in the same volume of buffer. Unless otherwise stated, all procedures were as previously described [34]. The following primary antibodies were used in dilutions of 1:20: anti-CR1 clone E11, anti-CD55 clone IA10, and isotype controls for each (Becton-Dickinson, Belgium). A secondary fluorescein isothiocyanate (FITC)-conjugated goat anti-mouse IgG (Becton-Dickinson, San Diego, CA) was used at a dilution of 1:50.

Flow cytometry was carried out using a FACScan flow cytometer (Becton-Dickinson). Analysis was done using FCS Express v2.5 (De Novo Software, Los Angeles, CA). Red cells were gated on the basis of their forward and side scatter characteristics using logarithmic amplification. The median fluorescence intensity (MFI) of each sample was measured using logarithmic amplification. The MFI values for CR1 and CD55 were normalized to the mean of the MFI of the red cell standard using the formula

*CorrMFIs *= *MFIs *× *MFIcmean*/*MFIc*,

where '*CorrMFIs*' and '*MFIs*' are the corrected and uncorrected sample MFI respectively, '*MFIcmean*' is the mean of all the MFI values of the standard control, and '*MFIc*' is the mean of the control obtained in parallel with the sample. The number of molecules of CR1 per red cell was derived from a fluorescence standard curve created using cells with known CR1 numbers. Red cell anti-CD55 antibody binding capacities were derived from a standard curve created using beads of known antibody binding capacity (Bangs Lab, Fishers, IN) [35].

### Preparation of ICs

50 μl of 49 mg/ml rabbit anti-BSA (Sigma-Adrich, St. Louis, MO) and 3 μl of 5 mg/ml BSA-FITC (Accurate Chemical and Scientific Corp., Westbury, NY) were added to 950 μl of RPMI1640 (Sigma-Aldrich). This combination was noted to be the point of equivalence in preliminary experiments. The mixture was incubated at 37°C for 1 hr and overnight at 4°C. The next day, the IC preparation of soluble and insoluble IC was aliquoted and stored at -20°C.

### Measurement of C3b on red cells

All centrifugation steps were at × 500 g for 5 min. Rabbit polyclonal anti-C3a (negative control antibody) and anti-C3b (Accurate) were pre-adsorbed ×3 by adding a 1:50 dilution of antibody in phosphate buffered saline (PBS) pH 7.4 to an equal volume of packed pre-washed erythrocytes from the normal standard control. The cells were incubated for 1 hr at 37°C with constant rocking followed by centrifugation. The pre-adsorbed antibody was frozen at -20°C in single-use aliquots. 100 μl of pre-washed freshly thawed erythrocytes at 1% hematocrit in Alsever's buffer was added to wells of a 96-well plate and resuspended in 50 μl of pre-adsorbed rabbit anti-C3b, anti-C3a, or in PBS (unstained control), and incubated for 10 min at 37°C. After two washes in PBS, the cells were resuspended in 1:50 anti-rabbit PE (Sigma-Aldrich) for 30 min at room temperature, washed twice, and resuspended again in PBS. Acquisition was carried out as above. The %C3b-positive cells were calculated by Overton subtraction [36] of the baseline C3a histogram from the baseline C3b histogram.

### Measurement of IC binding capacity

For IC opsonization, 5 μl of stock IC or RPMI 1640 (unstained control) was incubated in a total volume of 100 μl containing 30% AB+ serum in wells of a 96-well plate. For a negative control, a separate set of wells contained IC plus 10 mM EDTA. Following incubation at 37°C for 30 min with constant rocking motion, 100 μl of 1% hematocrit suspension of freshly thawed red cells from each study participant or from a standard aparasitemic control in RPMI 1640 was added to each of the wells of the above 96-well plate. This was followed by further incubation for 30 min at 37°C. The erythrocytes were then washed twice in 200 μl of ice-cold RPMI 1640, resuspended in PBS containing 1% paraformaldehyde, and stored at 4°C until acquisition. After gating, the erythrocyte FITC fluorescence was measured using logarithmic amplification and the positive cutoff was set using the unstained cells. The percent of positive red cells (IC binding capacity) was calculated based on this cutoff. To control for day-to-day variation, the IC binding capacity was normalized to the mean IC binding capacity of the red cell standard used throughout using a formula similar to the one used for correction of CR1 and CD55 (above).

### Statistical analysis

Statistical analysis was carried out using SPSS v11.5 (SPSS Inc., Chicago, IL). The flow cytometry and parasite density data are presented graphically for each age group as box plots, where the box represents the boundaries between the 25^th ^and 75^th ^percentile, the line through the box represents the median, the whiskers the 10^th ^and 90^th ^percentile limits, and the dots are the outliers. Analysis of variance (ANOVA) was used to detect differences across age groups adjusting for factors and covariates as indicated in the figure legends. P values were adjusted for multiple comparisons using the Sidak correction. The independent samples *t*-test was used for comparisons of normal continuous data between two groups. The chi-square test was used to compare proportions across groups. The general linear model procedure was used to carry out univariate ANOVA and analysis of covariance (ANCOVA) to identify independent variables associated with the %C3b-positive red cells and the hemoglobin level. Age groups, red cell CR1, red cell CD55, parasite density, malaria status, and IC binding capacity were the independent predictor variables included in the models. %C3b-positive red cells were also used as an independent variable for the hemoglobin level. Categorical variables were transformed into dummy variables and the B coefficient for the last group set to '0' for reference. All tests were two-sided with α ≤ 0.05.

## Results

### Demographic and clinical characteristics of the study population

Four hundred and two potential participants or parents of children who met the inclusion criterion signed the consent form and were screened. Of these, 344 met none of the exclusion criteria, attended the screening sessions, and agreed to provide blood samples (Table 1). Blood samples could not be drawn in sufficient quantities from two individuals, making the final number of samples available for the study 342. All individuals who were parasitemic were treated for malaria and in most cases the blood sample was collected 2 weeks later. Table 2 summarizes the demographic characteristics of the enrolled participants that contributed samples. We observed no difference in the proportion of locations or villages represented across the groups (data not shown).

**Table 1:** Disposition of participants

	Enrolled (*N*)	Deferred to Next Screen		Excluded	
			
		*N*	Reason for Deferral	*N*	Reason for Exclusion
Screen 1	190	194	190- positive blood smear,	18	3- refused blood draw
			4- non-malaria fever		12- malnourished or immunocompromised
					2- pneumonia
					1- severe anemia
Screen 2	151	10	All positive smear	33	1- age cohort full
					32- no shows
Screen 3	3	0		7	4- no shows,
					3- age cohort full

Total	344*			58	

*Two individuals excluded due to insufficient sample.

**Table 2:** Population demographics

**Age Groups (Months)**	**Total *N***	**No. Female (%)**	**Median Age in Months (Range)**
0 to ≤ 6	30	17 (56.7)	2.6 (0.4 to 0.6)
>6 to ≤ 12	60	29 (48.3)	10.9 (6.1 to 12.0)
>12 to ≤ 18	34	13 (38.2)	14.2 (12.1 to 17.5)
>18 to ≤ 24	27	14 (51.9)	21.3 (18.3 to 23.7)
>24 to ≤ 36	31	14 (45.2)	28.6 (24.2 to 36.0)
>36 to ≤ 72	42	22 (52.4)	42.7 (36.8 to 70.5)
>72 to ≤ 144	39	17 (43.6)	99.2 (73.1 to 143.9)
>144 to ≤ 288	24	12 (50.0)	248.9 (145.1 to 288.0)
>288 to ≤ 540	55	31 (56.4)	393.4 (290.7 to 540.0)

Total	342	169 (49.4)	29.7 (0.4 to 540.0)

### Red cell CR1, CD55, and IC binding capacity

**Figure 1:**
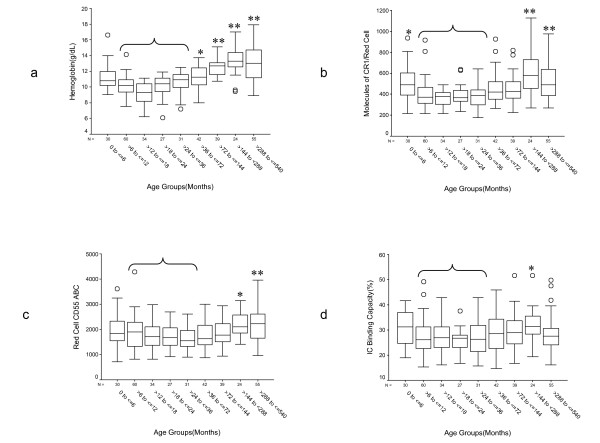
**Age-related changes in hemoglobin concentration, red cell CR1, CD55, and IC binding capacity**. For multiple comparison testing all groups were compared with the group formed by collapsing the groups ranging in age from >6 to ≤ 36 months (bracket). *P *values were adjusted using the Sidak correction for multiple comparisons. * = 0.01 <*P *≤ 0.05, ** = *P *< 0.01. a. Hemoglobin level. Unadjusted ANOVA for differences across groups *P *= 9.6 × 10^-38^. After adjustment for red cell CR1, red cell CD55, %C3b-positive red cells, malaria status, and parasite density, significant differences were found as indicated. b. Red cell CR1. Unadjusted ANOVA for differences across groups *P *= 1.5 × 10^-10^. After adjustment for malaria status and parasite density differences were found as indicated. c. Red cell CD55. Unadjusted ANOVA for differences across groups *P *= 6.1 × 10^-6^. After adjustment for malaria status and parasite density significant differences were found as indicated. ABC = Antibody binding capacity. d. binding capacity. Unadjusted ANOVA for differences across groups *P *= 0.004. After adjustment for malaria status and parasite density significant differences were found as indicated.

The nadir of hemoglobin level occurred in the >12 to ≤ 18 month group (Figure 1a). For multiple comparison testing we collapsed the age groups for age >6 to ≤ 36 months, which seemed to form a homogeneous subset in most analyses (data not shown), to reduce the number of comparisons and compared all other groups to this new group (Figure 1). The changes in hemoglobin level across groups were highly statistically significant. Although overall unadjusted changes in CR1, CD55, and IC binding capacity across age groups were statistically significant, only a limited number of comparisons were statistically significant after adjustment for malaria status and parasite density (Figure 1). Nonetheless, these findings confirm our previous results [37]. In the present study, children of between 6 and 36 months of age had the lowest red cell CR1 (Figure 1b) whereas children between 24 and 36 months of age had the lowest red cell CD55 (Figure 1c). The red cell IC binding capacity showed a pattern similar to that of CR1 but with a less marked rise in adulthood (Figure 1d).

### Red cell C3b, malaria prevalence, and parasite density

**Figure 2:**
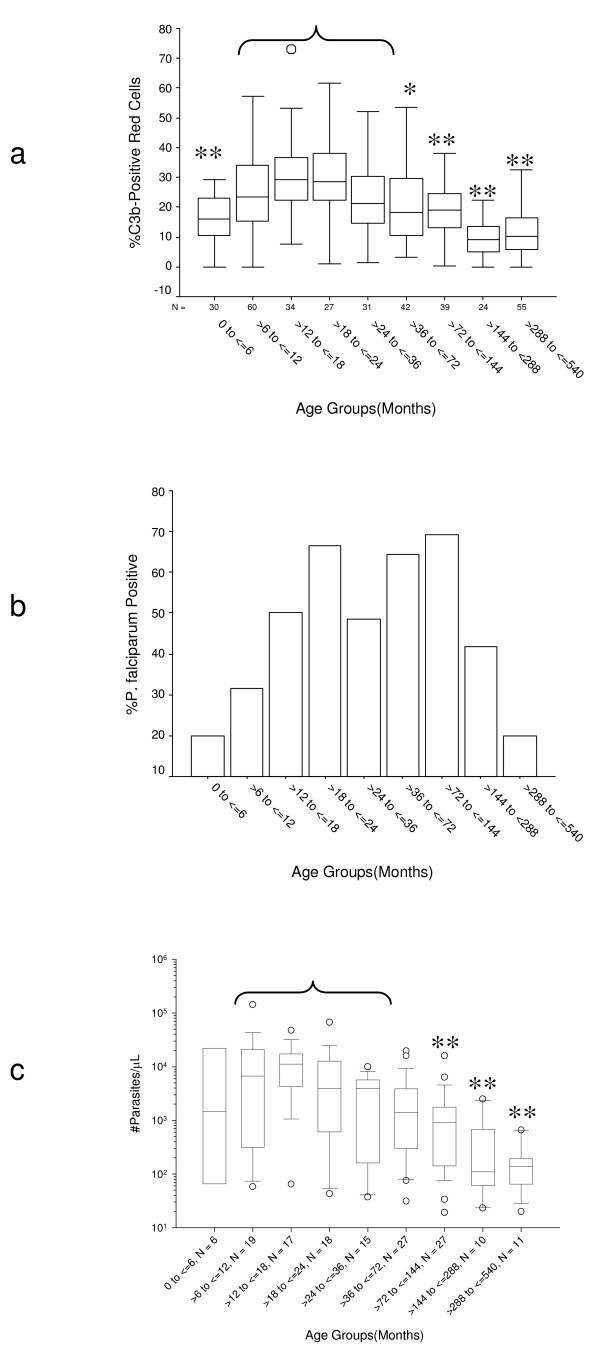
**Changes in %C3b-positive red cells, parasite prevalence, and parasite density with age**. a.%C3b-positive red cells by age. For multiple comparison testing age groups >6 to ≤ 36 months were collapsed into one. *P *values were obtained by one-way analysis of variance adjusting for red cell CR1, red cell CD55, IC binding capacity, malaria status, and parasite density.* = 0.01 <*P *≤ 0.05, ** = *P *< 0.01. Sidak correction for multiple comparisons was used. b. Prevalence of parasitemia by age group. c. Parasite density among malaria-positive individuals by age group. Parasite densities were Log_10_-transformed due to unequal variances between groups. Groups from >6 to ≤ 36 months of age were collapsed into one group and unadjusted comparisons between it and all other groups group were carried out. Sidak correction for multiple comparisons was used; ** = *P *< 0.01.

The %C3b-positive red cells increased from birth, peaked between 12 and 24 months of age and then decreased into adulthood (Figure 2a). Significant statistical differences in %C3b-positive red cells were observed when comparing children >6 to ≤ 36 months with other age groups (Figure 1a). The prevalence of *P. falciparum *malaria and the parasite density both rose and peaked within this age group. However, the prevalence of *P. falciparum *malaria (Figure 2b) and the distribution of parasite densities among malaria-positive (heretofore referred to as malaria-treated) individuals (Figure 2c) were discordant. The parasite prevalence increased from birth to 18 months, and peaked between 18 and 24 months, remaining high up to age 12 years, and decreasing thereafter (Figure 2b). A similar age distribution of parasite prevalence was observed when all the individuals screened were included, except that the prevalence was higher across all groups (data not shown). On the other hand, the parasite densities among malaria-treated individuals peaked between 12 and 18 months of age and were highest between 6 and 36 months (Figure 2c).

**Figure 3:**
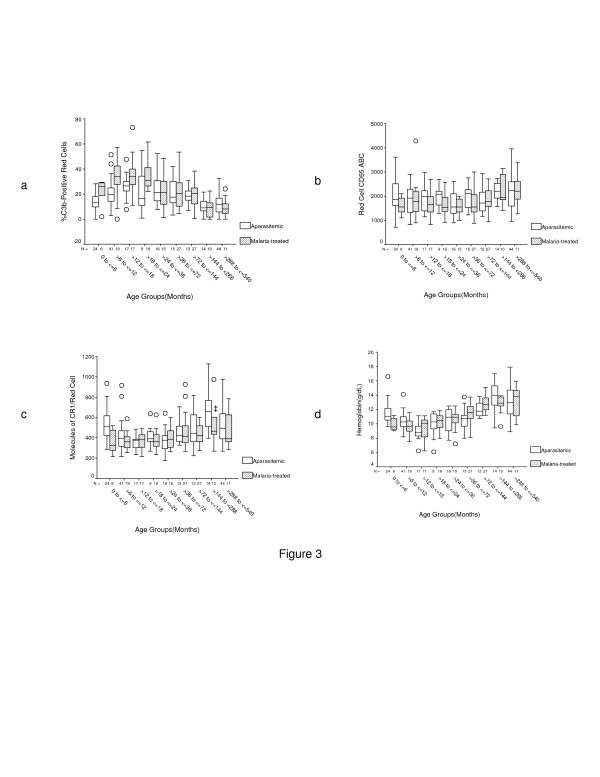
**Changes in %C3b-positive red cells, CR1, CD55, and hemoglobin according to malaria status**. a. %C3b-positive red cells. Unadjusted ANOVA for the difference in %C3b-positive red cells across groups *P*(malaria-treated) = 2.5 × 10^-11^, *P*(aparasitemic) = 1.2 × 10^-7^. Independent samples *t*-test for the comparison between malaria-treated and aparasitemic individuals for the indicated age groups, *P = 0.04, ^†^*P *= 2.8 × 10^-4^, ^‡^*P *= 0.09, ^¶^*P *= 0.03. b. Red cell CD55. Unadjusted ANOVA for the difference in CD55 across groups, *P*(malaria-treated) = 0.01 and *P*(aparasitemic) = 0.05. ABC = Antibody binding capacity. c. Red cell CR1. Unadjusted ANOVA for the difference in CR1 across groups, *P*(malaria-treated) = 0.02 and P(aparasitemic) = 1.9 × 10^-6^. Independent samples *t*-test for the comparison between malaria-treated and aparasitemic individuals for the indicated age groups, *P = 0.01, ^†^*P *= 0.58, ^‡^*P *= 0.09. d. Hemoglobin level. Unadjusted ANOVA for the difference in Hgb level across groups, *P*(malaria-treated) = 9.8 × 10^-19^, *P*(aparasitemic) = 3.8 × 10^-20^. Independent samples *t*-test for the comparison between malaria-treated and aparasitemic individuals for the indicated age groups, *P = 0.02, ^†^*P *= 0.05, ^‡^*P *= 0.03, ^¶^*P *= 0.11.

When participants were subcategorized according to the presence or absence of *P. falciparum *at enrollment (Figure 3) we observed that there was an age-dependent distribution of %C3b-positive red cells both in malaria-treated and aparasitemic individuals that was more marked in the former (Figure 3a). In addition, malaria-treated children of age ≤ 24 months had a greater proportion of C3b-positive red cells than their aparasitemic counterparts, although the difference did not reach statistical significance in the >12 to ≤ 18 month group. It was surprising to see that malaria-treated children of age ≤ 6 months (*n *= 6) had a high level of %C3b-positive red cells, but this was explained by relatively low red cell CR1 in these individuals. With few exceptions there were no significant differences in levels of CR1, CD55, and hemoglobin between malaria-treated and aparasitemic volunteers (Figures 3b to 3d).

### Analysis of variance to detect associations

We carried out analysis of variance to identify the factors most strongly associated with the %C3b-positive red cells and with the hemoglobin level in all the samples and in the subgroups of aparasitemic and malaria-treated participants (Tables 3 and 4). For the C3b analysis, terms for age groups, red cell CR1, IC binding capacity, malaria status (included for all the samples only), parasite density at enrollment, and red cell CD55 were used as independent variables. There were no significant interactions between CR1 and CD55, between CR1 or CD55 and age, or parasite density and other factors. The unadjusted and adjusted (all variables entered) results are presented in Table 3. Age was the most influential factor in all the analyses. Inclusion in the age group >6 to ≤ 36 months always had the strongest positive effect on C3b deposition, as seen from its larger correlation coefficient and positive B coefficient. In the unadjusted analysis, CR1 and CD55 were more influential than malaria status, parasite density, or IC binding capacity both in the global and in the subgroup analyses. After adjustment for all the confounding effects, malaria status and CD55 made the most significant contribution after age when all the samples were included, while CR1 showed a trend towards significance (*P *= 0.07). For the adjusted analysis of samples from malaria-treated individuals, age was the only statistically significant factor followed by CR1, which showed a trend towards significance (*P *= 0.09). Of interest, parasite density was not important in either analysis. Lastly, in the adjusted analysis of samples from malaria-negative individuals, CD55 and IC binding capacity made the strongest contribution after age.

**Table 3:** Univariate analysis of variance for %C3b-positive red cells as dependent variable

		Unadjusted					Adjusted				
		
Groups	Independent Variables	*F*	*B *(SE)	95% CI of *B*	*η*^2^*p*	*P*	*F*	*B *(SE)	95% CI of *B*	*η*^2^*p*	*P*
All	Age Groups (months)	21.8	--	--	0.245	5.9 × 10^-19^	11.6	--	--	0.149	2.5 × 10^-10^
*N* = 342	0 to ≤ 6	--	4.13 (2.53)	-0.85 to 9.10	0.008	0.10	--	3.29 (2.45)	-1.38 to 8.29	0.006	0.18
	>6 to ≤ 36	--	15.08 (1.75)	11.63 to 18.53	0.180	3.1 × 10^-16^	--	11.61 (1.81)	8.05 to 15.18	0.111	1.8 × 10^-10^
	>36 to ≤ 72	--	9.17 (2.28)	4.68 to 13.66	0.046	7.3 × 10^-5^	--	5.99 (2.30)	1.48 to 10.51	0.020	0.01
	>72 to ≤ 144	--	7.64 (2.33)	3.05 to 12.23	0.031	1.2 × 10^-3^	--	4.61 (2.36)	-0.03 to 9.24	0.011	0.07
	>144 to ≤ 288	--	-1.64 (2.72)	-7.00 to 3.72	0.001	0.55	--	-1.38 (2.68)	-6.64 to 3.88	0.001	0.54
	>288 to ≤ 540*	--	--	--	--	--	--	--	--	--	--
	CR1	46.1	-0.026 (0.004)	-0.034 to -0.019	0.119	5.1 × 10^-11^	3.2	-0.008 (0.004)	-0.016 to 0.001	0.010	0.07
	CD55	35.3	-0.007 (0.001)	-0.009 to -0.004	0.094	7.0 × 10^-9^	5.5	-0.003 (0.001)	-4.8 × 10^-3 ^to -0.4 × 10^-3^	0.016	0.02
	Malaria Negative^†^	17.8	-5.7 (1.4)	-8.4 to -3.1	0.050	3.2 × 10^-5^	6.4	-3.34 (1.32)	-5.93 to -0.74	0.019	0.01
	Parasite Density	8.8	1.9 × 10^-4 ^(0.6 × 10^-4^)	0.6 × 10^-4 ^to 3.2 × 10^-4^	0.025	3.2 × 10^-3^	0.5	4.1 × 10^-5 ^(5.9 × 10^-5^)	-0.8 × 10^-4 ^to 1.6 × 10^-4^	0.001	0.49
	ICBC	12.9	-0.358 (0.100)	-0.554 to -0.162	0.037	3.8 × 10^-4^	2.0	-0.130 (0.092)	-0.311 to 0.050	0.006	0.16

Malaria-treated	Age Groups (months)	12.7	--	--	0.306	3.2 × 10^-10^	9.0	--	--	0.244	1.8 × 10^-7^
*N* = 150	0 to ≤ 6	--	12.36 (6.06)	0.38 to 24.35	0.028	0.04	--	10.08 (6.38)	-2.53 to 22.68	0.018	0.12
	>6 to ≤ 36	--	21.48 (3.88)	13.81 to 29.15	0.175	1.4 × 10^-7^	--	20.00 (4.13)	11.83 to 28.18	0.143	3.4 × 10^-6^
	>36 to ≤ 72	--	10.68 (4.28)	2.23 to 19.13	0.042	0.01	--	10.01 (4.42)	1.28 to 18.73	0.035	0.03
	>72 to ≤ 144	--	9.49 (4.28)	1.04 to 17.94	0.033	0.02	--	8.25 (4.43)	-0.51 to 17.00	0.024	0.07
	>144 to ≤ 288	--	-0.78 (5.22)	-11.11 to 9.53	1.6 × 10^-4^	0.88	--	-0.69 (5.26)	-11.10 to 9.72	1.2 × 10^-4^	0.90
	>288 to ≤ 540*	--	--	--		--	--	--	--	--	--
	CR1	13.7	-0.030 (0.008)	-0.046 to -0.014	0.085	3.0 × 10^-4^	3.0	-0.015 (0.009)	-.032 to 0.002	0.021	0.09
	CD55	7.2	-0.005 (0.002)	-0.009 to -0.001	0.047	7.9 × 10^-3^	0.01	2.5 × 10^-4 ^(21.1 × 10^-4^)	-0.004 to 0.004	1.0 × 10^-4^	0.91
	Parasite Density	2.6	1.2 × 10^-4 ^(0.8 × 10^-4^)	-0.3 × 10^-4 ^to 2.7 × 10^-4^	0.017	0.11	0.1	-1.9 × 10^-5 ^(6.8 × 10^-5^)	-1.5 × 10^-4 ^to 1.1 × 10^-4^	0.001	0.78
	ICBC	0.2	-0.069 (0.171)	-0.408 to 0.269	0.001	0.69	0.3	0.085 (0.155)	-0.222 to 0.392	0.002	0.59

Malaria Negative	Age Groups (months)	9.9	--	--	0.210	2.2 × 10^-8^	4.8	--	--	0.116	6.3 × 10^-5^
*N* = 192	0 to ≤ 6	--	2.07 (2.50)	-2.87 to 7.00	0.004	0.41	--	1.76 (2.35)	-2.93 to 6.34	0.003	0.47
	>6 to ≤ 36	--	10.80 (1.84)	7.17 to 14.43	0.156	2.0 × 10^-8^	--	7.93 (1.82)	4.71 to 11.78	0.094	7.9 × 10^-6^
	>36 to ≤ 72	--	9.23 (2.95)	3.41 to 15.06	0.050	0.002	--	7.76 (2.78)	2.49 to 13.43	0.041	4.6 × 10^-3^
	>72 to ≤ 144	--	7.09 (3.21)	0.75 to 13.43	0.025	0.03	--	4.66 (3.05)	-1.44 to 10.60	0.013	0.13
	>144 to ≤ 288	--	-1.42 (3.03)	-7.39 to 4.56	0.001	0.64	--	0.33 (2.91)	-5.83 to 5.45	7.0 × 10^-5^	0.95
	>288 to ≤ 540*	--	--	--	--	--	--	--	--	--	--
	CR1	29.4	-0.022 (0.004)	-0.030 to -0.014	0.134	1.8 × 10^-7^	1.0	-0.004 (0.005)	-0.014 to 0.005	0.005	0.33
	CD55	27.0	-0.006 (0.001)	-0.009 to -0.004	0.124	5.2 × 10^-7^	8.6	-0.004 (0.001)	-0.006 to -0.001	0.045	3.8 × 10^-3^
	ICBC	26.9	-0.564 (0.109)	-0.779 to -0.350	0.124	5.4 × 10^-7^	9.0	-0.326 (0.109)	-0.540 to -0.111	0.047	3.1 × 10^-3^

*B for this group set to '0' as reference.

^†^*B *for malaria-treated group was set to '0' as reference.

*η*^2^*p *= Partial Eta squared or coefficient of non-linear correlation.

ICBC = IC binding capacity.

**Table 4:** Univariate analysis of variance for hemoglobin as dependent variable

		Unadjusted					Adjusted				
		
Groups	Independent Variables	*F*	*B *(SE)	95% CI of *B*	*η*^2^*p*	*P*	*F*	*B *(SE)	95% CI of *B*	*η*^2^*p*	*P*
All	Age Groups (months)	48.5	--	--	0.419	9.6 × 10^-38^	26.5	--	--	0.287	1.5 × 10^-22^
*N* = 342	0 to ≤ 6	--	-1.94 (0.36)	-2.64 to -1.23	0.080	1.2 × 10^-7^	--	-1.86 (0.34)	-2.54 to -1.18	0.080	1.5 × 10^-7^
	>6 to ≤ 36	--	-3.13 (0.25)	-3.62 to -2.64	0.321	4.7 × 10^-30^	--	-2.59 (0.27)	-3.13 to -2.07	0.219	7.3 × 10^-19^
	>36 to ≤ 72	--	-1.94 (0.32)	-2.58 to -1.31	0.097	5.0 × 10^-9^	--	-1.72 (0.33)	-2.36 to -1.08	0.078	2.3 × 10^-7^
	>72 to ≤ 144	--	-0.54 (0.33)	-1.19 to 0.11	0.008	0.10	--	-0.35 (0.33)	-1.01 to 0.30	0.003	0.29
	>144 to ≤ 288	--	0.20 (0.39)	-0.55 to 0.96	0.001	0.60	--	-0.11 (0.38)	-0.85 to 0.63	2.6 × 10^-4^	0.77
	>288 to ≤ 540*	--	--	--	--	--	--	--	--	--	--
	CR1	63.3	0.005 (0.001)	0.004 to 0.006	0.157	2.7 × 10^-14^	20.4	0.003 (0.001)	0.002 to 0.004	0.058	8.7 × 10^-6^
	CD55	10.3	5.9 × 10^-4 ^(1.8 × 10^-4^)	2.3 × 10^-4 ^to 9.5 × 10^-4^	0.029	1.5 × 10^-3^	5.9	-3.8 × 10^-4 ^(1.6 × 10^-4^)	-6.9 × 10^-4 ^to -0.7 × 10^-4^	0.018	0.02
	Malaria Status^†^	1.2	0.246 (0.224)	-0.195 to 0.686	0.004	0.27	0.3	-0.103 (0.187)	-0.471 to 0.265	0.001	0.58
	Parasite Density	4.9	-2.3 × 10^-5 ^(1.0 × 10^-5^)	-4.3 × 10^-5 ^to -0.3 × 10^-5^	0.014	0.03	1.1 × 10^-5^	0.3 × 10^-7 ^(82.7 × 10^-7^)	-1.6 × 10^-5 ^to 1.6 × 10^-5^	3.5 × 10^-8^	1.00
	ICBC	11.9	0.056 (0.016)	0.024 to 0.087	0.034	6.3 × 10^-4^	0.02	0.002 (0.013)	-0.024 to 0.027	6.0 × 10^-5^	0.89
	%C3b-positive Red Cells	77.6	-0.070 (0.008)	-0.085 to -0.054	0.186	6.6 × 10^-17^	10.5	-0.025 (0.008)	-0.040 to -0.010	0.031	1.3 × 10^-3^

Malaria-treated	Age Groups(months)	27.2	--	--	0.486	2.7 × 10^-19^	15.3	--	--	0.355	6.0 × 10^-12^
*N* = 150	0 to ≤ 6	--	-3.09 (0.68)	-4.42 to -1.75	0.127	1.1 × 10^-5^	--	-2.74 (0.68)	-4.09 to -1.39	0.103	1.0 × 10^-4^
	>6 to ≤ 36	--	-2.98 (0.43)	-3.83 to -2.12	0.247	1.7 × 10^-10^	--	-2.54 (0.48)	-3.48 to -1.60	0.170	3.7 × 10^-7^
	>36 to ≤ 72	--	-1.53 (0.48)	-2.47 to -0.59	0.067	1.7 × 10^-3^	--	-1.45 (0.48)	-2.39 to -0.50	0.062	2.9 × 10^-3^
	>72 to ≤ 144	--	-0.12 (0.48)	-1.06 to 0.82	4.5 × 10^-4^	0.80	--	-0.03 (0.48)	-0.91 to 0.97	3.0 × 10^-5^	0.95
	>144 to ≤ 288	--	-0.31 (0.58)	-1.46 to 0.85	0.002	0.60	--	-0.46 (0.56)	-1.56 to 0.65	0.005	0.41
	>288 to ≤ 540*	--	--	--		--	--	--	--	--	--
	CR1	28.2	0.005 (0.001)	0.003 to 0.007	0.160	3.9 × 10^-7^	13.0	0.003 (0.001)	0.002 to 0.005	0.085	4.4 × 10^-4^
	CD55	7.2	7.0 × 10^-4 ^(2.6 × 10^-4^)	1.9 × 10^-4 ^to 12.2 × 10^-4^	0.047	8.1 × 10^-3^	2.0	-3.2 × 10^-4 ^(2.2 × 10^-4^)	-7.6 × 10^-4 ^to 1.3 × 10^-4^	0.014	0.16
	Parasite Density	5.0	-2.2 × 10^-5 ^(1.0 × 10^-5^)	-4.1 × 10^-5 ^to -0.3 × 10^-5^	0.033	0.03	0.002	3.2 × 10^-7 ^(0.7 × 10^-7^)	-1.4 × 10^-5 ^to 1.5 × 10^-5^	1.4 × 10^-5^	0.96
	ICBC	3.2	0.039 (0.022)	0.004 to 0.083	0.021	0.08	0.3	0.008 (0.017)	-0.024 to 0.041	0.002	0.61
	%C3b-positive Red Cells	34.9	-0.057 (0.010)	-0.075 to -0.038	0.191	2.3 × 10^-8^	1.9	-0.012 (0.009)	-0.030 to 0.005	0.014	0.17

Malaria Negative	Age Groups (months)	26.4	--	--	0.415	4.4 × 10^-20^	14.4	--	--	0.284	7.0 × 10^-12^
*N* = 192	0 to ≤ 6	--	-1.65 (0.44)	-2.51 to -0.79	0.072	2.1 × 10^-4^	--	-1.63 (0.42)	-2.46 to -0.80	0.077	1.4 × 10^-4^
	>6 to ≤ 36	--	-3.15 (0.32)	-3.78 to -2.52	0.342	1.2 × 10^-18^	--	-2.62 (0.34)	-3.30 to -1.95	0.246	8.0 × 10^-13^
	>36 to ≤ 72	--	-2.41 (0.51)	-3.43 to -1.40	0.106	5.2 × 10^-6^	--	-1.97 (0.51)	-2.97 to -0.97	0.077	1.4 × 10^-4^
	>72 to ≤ 144	--	-1.15 (0.56)	-2.25 to -0.04	0.022	0.04	--	-0.98 (0.55)	-2.06 to 0.10	0.017	0.07
	>144 to ≤ 288	--	0.65 (0.53)	-0.39 to 1.69	0.008	0.22	--	0.31 (0.52)	-0.71 to 1.33	0.002	0.55
	>288 to ≤ 540*	--	--	--	--	--	--	--	--	--	--
	CR1	33.9	0.005 (0.001)	0.003 to 0.006	0.151	2.4 × 10^-8^	7.6	0.002 (0.001)	0.001 to 0.004	0.040	0.01
	CD55	3.5	4.9 × 10^-4 ^(2.6 × 10^-4^)	-0.2 × 10^-4 ^to 10.0 × 10^-4^	0.018	0.06	5.0	-4.9 × 10^-4 ^(2.2 × 10^-4^)	-9.3 × 10^-4 ^to -0.6 × 10^-4^	0.027	0.03
	ICBC	8.6	0.068 (0.023)	0.022 to 0.113	0.043	3.7 × 10^-3^	0.4	-0.012 (0.020)	-0.052 to 0.027	0.002	0.53
	%C3b-positive Red Cells	46.3	-0.090 (0.013)	-0.116 to -0.064	0.196	1.3 × 10^-10^	10.7	-0.043 (0.013)	-0.069 to -0.017	0.056	1.3 × 10^-3^

*B for this group set to '0' as reference.

^†^*B *for malaria-treated group was set to '0' as reference.

*η*^2^*p *= Partial Eta squared or coefficient of non-linear correlation.

ICBC = IC binding capacity.

For the analysis of factors associated with the hemoglobin level (Table 4), once again age made the strongest contribution both in the unadjusted and adjusted analyses. Of all the age groups, the >6 to ≤ 36 months group also had the strongest negative effect on the hemoglobin level. In the unadjusted analysis, %C3b-positive red cells, parasite density, and red cell CR1 always exerted a significant influence on hemoglobin level. The IC binding capacity made significant contributions in the unadjusted analysis of all the samples and the samples from malaria negative individuals. In the adjusted analysis, in addition to age, red cell CR1, %C3b-positive red cells, and CD55 made a significant contribution to the hemoglobin level using all the samples and the samples from individuals who were negative for malaria. Interestingly, CD55 among aparasitemic individuals had a negative, although very small, B coefficient, meaning that in this group CD55 seemed to be associated with declining hemoglobin level. For the analysis that included samples from malaria-treated individuals, red cell CR1 and age exerted the greatest influence on the hemoglobin level.

## Discussion

Severe anemia due to *P. falciparum *occurs principally in young children and is most common in areas of intense malaria transmission [4,5,38]. Cerebral malaria, on the other hand, occurs in older children and adults in areas of low transmission, and the incidence can increase with age [5,39]. Identifying the mechanisms underlying these disparities in age-related susceptibilities is critical to understanding the pathogenesis of severe malaria. We previously reported that the levels of red cell complement regulatory proteins CR1 and CD55, which protect red cells from autologous complement attack, vary with age, being high in neonates, decreasing after 6 months and increasing some time after into adulthood [40]. This pattern of expression is independent of malaria infection and of the ethnic background of the population [41]. Our primary objective was to determine whether low levels of red cell complement regulatory proteins are associated with increased C3b deposition on red cells and, secondarily, with low hemoglobin in a malaria-endemic area. Because we have previously observed that individuals with malaria have lower levels of red cell complement regulatory proteins [13,42], we controlled for the effect of parasitemia on this parameter by administering anti-malarial treatment to malaria-positive participants and collecting the blood sample only when they were smear negative. Restrictions on the amount of blood that can be drawn from very young children limited the number of blood draws within our time frame to one.

The relationship between red cell C3b and red cell complement regulatory protein expression seemed complex (Figures 1 and 2a). Red cell CR1 appeared to be more age-dependent than CD55 or the IC binding capacity. The peak of %C3b-positive red cells coincided with the nadir of red cell CR1 (Figures 1b and 2a) suggesting a stronger relationship between C3b and CR1 than between C3b and CD55 or the IC binding capacity. However, in the overall ANOVA model (Table 3) CD55 made a stronger contribution. This could be explained by the fact that CD55 seemed to be more important in preventing C3b deposition in malaria-negative individuals, who were more numerous in the study group. Although not statistically significant (*P *= 0.09), CR1 appeared to be more important than CD55 in protecting red cells from C3b deposition during malaria infection (Table 3). These observations point to distinct mechanisms of action between CR1 and CD55. Since studies have shown that there is active complement activation during malaria infection [9,43], our observations suggest that CR1 is more effective than CD55 at preventing C3b deposition on cells.

The presence of parasitemia seemed to be a more important contributor to C3b deposition than the degree of parasitemia. This is somewhat surprising since although parasite density peaked at an earlier age than parasite prevalence (Figures 2b and 2c), both parameters peaked within the same age range as the peak for %C3b-positive red cells (Figure 2a). However, malaria-treated individuals ≤ 24 months of age tended to have higher %C3b-positive red cells than malaria-negative individuals, regardless of the level of parasitemia (Figures 2c and 3a), suggesting that the level of parasitemia is less important. Although we cannot exclude the possibility that malaria treatment may have exacerbated C3b deposition on red cells, and thus biased our results, we have observed C3b deposition on red cells of acutely infected children prior to treatment in other studies [44]. Thus, the C3b deposition on red cells of children who were treated for malaria is likely residual from the original infection.

It is interesting that increased C3b deposition was also seen on red cells of children with low red cell complement regulatory protein but without malaria infection, suggesting that there is a defect in the normal regulatory mechanisms during this age that prevent spontaneous complement activation. Alternatively, C3b on aparasitemic individuals may have been residual from recent previous malaria infections or infections with other agents. It is also possible that some or many of these individuals have levels of parasitemia below the limit of detection of microscopy.

The question of whether parasite prevalence or parasite density is a contributor to uninfected red cell destruction has important ramifications for the understanding of the pathogenesis of severe anemia during malaria. Current belief is that natural acquired immunity does not sterilize but suppresses the level of parasitemia and, therefore, parasite density is a reflection of the level of immunity [45]. Hence, if malaria prevalence is a function of exposure only, with no role for acquired immunity, at least early on, this would suggest that the age specificity of severe anemia due to malaria is being driven solely by the physiologic changes in the ability of children to regulate complement activation, namely age-related declines in red cell complement regulators. In this case, one would predict that there would be no relationship between severe anemia and the level of parasitemia, as we have found in previous studies [13,46,47]. On the other hand, if changes in parasite prevalence are being driven by acquisition and loss of immunity then obviously immunity would play a role, but in this case one would expect parasite density to be an important factor as well.

The most important variables that influenced the hemoglobin level in this study were age, red cell CR1, %C3b-positive red cells, and red cell CD55 (Table 4). Interestingly, the effect of CD55 on hemoglobin was always negative. The most likely reason is because as CD55 decreased to a nadir at age >24 to ≤ 36 months the hemoglobin increased (Figures 2a and 2c). These observations are consistent with the role of C3b in the destruction of red cells and with the more important role of CR1 over CD55 in the protection of red cells from complement activation. In the analysis of samples from malaria-treated individuals, only CR1 and age made significant contributions. The absence of %C3b as an important factor here is surprising given the high proportion of C3b-positive red cells associated with low hemoglobin in malaria-treated individuals (Figures 3a and 3d), and suggests that even in the setting of high C3b deposition high red cell CR1 may be protective. The failure of parasite density and malaria status to make significant contributions to the hemoglobin level probably reflects the fact that malaria treatment eliminated any difference in the hemoglobin level between malaria-treated and aparasitemic participants (data not shown). Therefore, the model presented here is free of the direct effects of the parasite on the destruction of red cells but retains the indirect effects of complement-mediated mechanisms.

The two ANOVA models (Tables 3 and 4) suggest the importance of age as the strongest factor determining C3b deposition on red cells and the hemoglobin level. The implication of this finding for complement activation is that quite possibly there still remain to be identified other age-related factors that regulate complement activation and deposition on red cells. The relationship between age and hemoglobin level is probably multifactorial and likely includes factors such as diet, and iron deficiency as well as the contribution of age effects on complement regulation.

Our data suggest that children >6 to ≤ 36 months of age who develop malaria are at high risk of uninfected red cell destruction due to ineffective complement regulation. They have relatively low red cell CR1 that may be insufficient to control complement activation on cell surfaces. On the other side of this equation is the contribution of macrophage activation, which should also be considered. Macrophages can be activated by the malaria pigment hemozoin as well as by malaria GPI, leading to increased production of pro-inflammatory cytokines which can upregulate the expression of complement receptors leading to phagocytosis [48-50]. In addition, several studies have documented an inverse relationship between TNF-α production, in response to malaria infection, and age [51,52]. Thus, in addition to high red cell C3b, parasitemic children ≤ 24 months of age may have overstimulated macrophages, making their red cells more susceptible to erythrophagocytosis and severe anemia.

## Conclusion

These results suggest that increasing malaria prevalence among children >6 to ≤ 36 months of age in western Kenya together with low red cell CR1 and CD55 levels result in increased C3b deposition on red cells leading to increased red cell destruction by phagocytosis. The strong contribution of age to C3b deposition suggests that there are still additional unidentified age-related factors that increase the susceptibility to C3b deposition on red cells and red cell destruction.

## Abbreviations

ANCOVA: analysis of covariance; ANOVA: analysis of variance; DAF: decay accelerating factor; EDTA: ethylenediaminetetraacetic acid; FITC: fluorescein isothiocyanate; GPI: glycosyl-phosphatidyl-inositol; IC: immune complex; MFI: median fluorescence intensity; PBS: phosphate buffered saline.

## Competing interests

The authors declare that they have no competing interests.

## Authors' contributions

COO carried out flow cytometry to measure the red cell C3b. WO supervised the recruitment and evaluation of the study participants. CA supervised the measurement of clinical laboratory parameters. MMO carried out flow cytometry to measure red cell CR1, CD55, and IC binding capacity. JAS and WO designed the study and wrote the protocol. COO and JAS drafted the manuscript which was reviewed and approved by all.

## Pre-publication history

The pre-publication history for this paper can be accessed here:


